# Role of Active Video Games in Blood Pressure Management Among Children and Young Adults: Systematic Review and Meta-Analysis

**DOI:** 10.2196/75000

**Published:** 2025-08-19

**Authors:** Hao Zhu, Keith Tsz-Suen Tung, Hung-kwan So, Parco M Siu, Ian Chi Kei Wong, Jason C Yam, Joanna Yuet-ling Tung, Yih-kuen Jan, Li He, Patrick Ip

**Affiliations:** 1 Department of Paediatrics and Adolescent Medicine, Li Ka Shing Faculty of Medicine, University of Hong Kong, Room 115, 1/F, New Clinical Building, 102 Pokfulam Road, Queen Mary Hospital, Hong Kong SAR, 999077, China (Hong Kong), 852 22554090; 2 Division of Kinesiology, School of Public Health, Li Ka Shing Faculty of Medicine, University of Hong Kong, Hong Kong SAR, China (Hong Kong); 3 Department of Pharmacology and Pharmacy, Li Ka Shing Faculty of Medicine, University of Hong Kong, Hong Kong SAR, China (Hong Kong); 4 Department of Ophthalmology and Visual Sciences, Chinese University of Hong Kong, Hong Kong SAR, China (Hong Kong); 5 Department of Paediatrics and Adolescent Medicine, Hong Kong Children’s Hospital, Hong Kong SAR, China (Hong Kong); 6 Department of Health and Kinesiology, University of Illinois Urbana-Champaign, Urbana, IL, United States; 7 School of Physical Education and Sports, Beijing Normal University, Beijing, China

**Keywords:** active video games, systolic blood pressure, diastolic blood pressure, children, young adults, PRISMA, Preferred Reporting Items for Systematic Reviews and Meta-Analyses

## Abstract

**Background:**

The significant association between blood pressure (BP) in children and young adulthood and risks of cardiovascular diseases in adulthood highlights the critical need for early BP control. While lifestyle modifications such as increased physical exercise have proven effective, traditional exercise forms always suffer from low motivation and adherence. Active video games (AVGs), combining exercise with engaging gameplay, may present a promising alternative for managing BP in children and young adults.

**Objective:**

This study aims to evaluate the effectiveness of AVGs in managing BP among the population aged 6 to 25 years.

**Methods:**

Following the PRISMA (Preferred Reporting Items for Systematic Reviews and Meta-Analyses) guideline, this study retrieved and screened publications archived in the 4 databases (Web of Science, Cochrane Library, PubMed, and Embase) and the registration (ClinicalTrials.gov) up to December 30, 2024. Eligible studies were defined as interventional trials involving participants aged 6 to 25 years, using AVGs as one of the intervention protocols, and reporting BP outcomes. Studies were excluded if they involved participants with heart diseases, combined AVGs protocol with other intervention components, limited outcomes to immediate postgame BP, or included only control groups that received additional physical activity interventions. Depending on the heterogeneity among included trials, random-effects or fixed-effects models were selected to pool the effect sizes of individual trials, with 95% CIs. The risk of bias was assessed using the Cochrane Risk of Bias tool for controlled trials and the Methodological Index for Non-Randomized Studies for prepost design. Sensitivity analyses were performed to evaluate result robustness, while Egger tests investigated publication bias.

**Results:**

A total of 17 trials from 16 studies, involving 503 participants who are normotensive, were included in this study. The analysis showed that AVGs significantly reduced systolic blood pressure (standardized mean difference=−0.50, *P*<.001, 95% CIs −0.80 to −0.20) and increased diastolic blood pressure (standardized mean difference=0.23, *P*=.03, 95% CIs 0.02 to 0.44) in children younger than 18 years, with the GRADE (Grading of Recommendations Assessment, Development and Evaluation) indicating the certainties of evidence as low for systolic blood pressure and moderate for diastolic blood pressure.

**Conclusions:**

These findings shed light on the cardiovascular benefits of AVGs in children younger than 18 years, underscoring their potential to improve vascular elasticity while maintaining organ perfusion. However, considering the limitations arising from small sample sizes, as well as inadequate allocation concealment and blinding in the included studies, these findings should be interpreted with caution.

## Introduction

### Background

Cardiovascular diseases (CVDs) remain a leading cause of morbidity and mortality worldwide, with blood pressure (BP) being a major modifiable risk factor. The increasing prevalence of elevated BP and hypertension among the younger generation underscores that BP management is no longer solely a concern for older adults [1,2]. Emerging evidence suggests that BP in children and young adulthood is strongly associated with the risk of developing CVDs in adulthood [3,4]. Cumulative exposure to high BP from adolescence to adulthood has been linked to myocardial dysfunction [5], accelerated vascular aging [6], and ultimately leads to target organ damage in later life [7]. This association highlights the critical importance of intervention strategies to manage BP effectively in early life. Early management can not only reduce immediate health risks but also help prevent long-term cardiovascular complications and break the intergenerational cycle of CVDs [8,9].

Lifestyle modifications, such as increased physical activity, dietary changes, and weight management, have always been the first-line treatment for managing BP in early life stages [10,11]. Among these, physical exercise has been widely recognized for its efficacy in improving cardiovascular health and reducing BP [12]. An increasing variety of exercise forms has been found to induce beneficial cardiovascular changes and help regulate BP [13-16]. However, these traditional means of exercise intervention are confronted with inherent problems including low exercise motivation and high dropout rates [17,18]. Repeated exposure to unappealing exercise content and formats can trigger emotional disengagement and lower exercise adherence in participants [19], ultimately leading to the loss of initial health benefits gained from exercise [20].

### Current Evidence

Against this backdrop, active video games (AVGs), also known as exergames, have emerged as a novel and innovative approach [21]. Previous studies have demonstrated that AVGs can increase habitual physical activity [22], promote energy expenditure [23], enhance cognitive flexibility [24], improve physical fitness [25], and achieve better weight management [26]. Unlike the negative cardiovascular impacts of sedentary-based video games [27], AVGs have also shown potential in improving cardiovascular health [28]. Some studies have found that AVGs can lower total cholesterol levels [29], improve microcirculation and vascular endothelial function [30], and show potential for regulating BP [31]. By integrating physical exertion into immersive video games, AVGs have captured the attention and engagement of the largest gaming demographic—children and young adults [32,33], making them a promising intervention option for managing BP that could be seamlessly integrated into the digital-centric lifestyles of today’s young people [34].

Despite the growing interest in AVGs, their specific effects on BP regulation remain inadequately explored. Some studies suggest that AVGs can effectively lower BP [21,30,31,35-38], while others have observed no significant BP reduction [28,29,39-44], and some even report BP elevation following AVGs [45]. These inconsistent findings highlight the necessity for a comprehensive review of the evidence. The lack of a quality systematic review will inevitably limit the applicability of AVGs in clinical practice and daily life.

### Objectives

This study aims to address this gap by systematically evaluating the current evidence on the effectiveness of AVGs in managing BP among individuals aged 6 to 25 years. This population consists of school-aged children (<18 years) and college young adults (18 to 25 years), who share homogeneous living environments due to their structured daily routines in educational settings. Additionally, the high receptivity to AVGs among this cohort of digital natives enables the findings to be directly translated into school-based health programs, establishing a “research-practice closed loop” for the seamless implementation of evidence-based strategies in educational contexts [32]. By comprehensively evaluating the resting systolic blood pressure (SBP) and diastolic blood pressure (DBP) responses after AVGs engagement, this study aims to explore the diverse effects of AVGs on BP during different phases of the cardiac cycle, thereby facilitating understanding of their potential value in vascular elasticity and organ perfusion.

## Methods

### Search Strategies and Selection Criteria

In this systematic review and meta-analysis, we developed search strategies for 4 selected databases (Web of Science, Cochrane Library, PubMed, and Embase) based on the PICOS (Participants, Intervention, Comparison, Outcome, and Study Design) framework and prior research [46,47]. Detailed search terms are presented in the Appendix S1 (P1 - 5) in Multimedia Appendix 1 [29,47-69]. Two researchers (HZ and Jingyi Zhou, MEd) independently searched these databases from their inception to December 30, 2024, while also searching records from registration (ClinicalTrials.gov) and references from relevant systematic reviews and meta-analyses (the information detailed in Appendix S1, P5 - 10 in Multimedia Appendix 1).

Retrieved studies were imported into EndNote X9 (Clarivate Analytics) software, and duplicates were removed. Subsequently, 2 researchers independently screened titles and abstracts and conducted full-text screening of the remaining studies based on the inclusion and exclusion criteria outlined in Table 1 to identify eligible studies. Discrepancies between the 2 researchers were resolved through discussion with another independent researcher (KTST).

**Table 1.** Inclusion and exclusion criteria for publications in this study.

Criteria	Inclusion criteria	Exclusion criteria
Population	The mean age of participants was older than 6 years but younger than 25 years	Participants were afflicted with heart diseases
Intervention	The intervention protocols encompassed AVGs^a^	The AVGs’ protocol was combined with additional intervention components
Control	For controlled trials: intervention strategies for the control group were clearly described For prepost trials, baseline and postintervention data were reported	All control groups received additional physical activity interventions
Outcome	Outcome measurements included at least one of SBP^b^ or DBP^c^	Outcome measurements were limited to immediate postintervention blood pressure The measurement procedures were not clearly described
Study design	Interventional studies	Interventional studies reporting outcomes qualitatively
Additional criteria	No language restrictions Publication date: from database inception to December 30, 2024	Studies with incomplete data after contacting authors for clarification

a AVG: active video game.

b SBP: systolic blood pressure.

c DBP: diastolic blood pressure.

### Data Extraction and Processing

The 2 primary outcomes of interest in this study were changes in resting SBP and DBP. For studies where BP was the primary outcome, all reported BP measurements were extracted directly from the results section. For studies where BP was a secondary or exploratory outcome, the methods section was carefully reviewed by the 2 researchers. Studies that did not clearly describe the BP measurement process were excluded from the analysis. After raw data from included trials were uniformly converted to mean (SD) using previously validated methods [70], the required changes were calculated according to the following formula [71,72]. For controlled trials, effect sizes were calculated by comparing changes between intervention and control groups. For prepost trials, effect sizes were represented by the changes in means and SDs from baseline to postintervention. In subsequent sections, we conducted a reanalysis of all controlled trials after excluding prepost trials as part of the sensitivity analyses to validate the robustness of the results.


 (1) $$Mean_{change}=Mean_{postexercise}-Mean_{baseline\;}$$

(2) $$SD_{change}=\;\sqrt{SD_{baseline}^{2}+SD_{postexercise}^{2}-2R\times SD_{baseline}\times SD_{postexercise}}$$

In these 2 formulas, mean_change_ and SD_change_ represent the changes in the mean and SD before and after intervention, respectively. Mean_postexercise_ and mean_baseline_ refer to the means after the intervention and at baseline. SD_baseline_ and SD_postexercise_ indicate the SDs before and after intervention, respectively. As none of the included studies provided correlation data on BP before and after AVGs, we adopted the R of 0.5, following the methodology established by Follmann et al [73]. This approach has been widely applied in many studies to investigate the effects of various exercises on BP [74-77].

Furthermore, the characteristics of studies were recorded, including basic information (first author’s name, publication year, country or region, and study design), participant demographics (sample size, gender, age, and BMI), and intervention characteristics (condition, duration, frequency, and time). For studies with missing data, their corresponding authors would be contacted via email at least 3 times. The data extraction and information entry above were independently performed and cross-verified 3 times by 2 researchers (HZ and Jingyi Zhou, MEd). Any persistent disagreements would be resolved through discussion with another independent researcher (KTST).

### Data Analysis

The statistical analyses were performed using STATA (version 18.0; Stata). Given the variation in measurement units and the relatively small sample sizes across the included studies, we used the standardized mean difference (SMD) and 95% CIs, corrected by Hedges g, to pool the overall effect size of all individual trials. Hedges g introduces a correction factor to Cohen *d*, which allows it to provide a more accurate effect size in situations involving small sample sizes [78].

Furthermore, the Q statistic and *I*² statistic were used to assess heterogeneity. If *I*² >50% or the *P* value for the Q statistic was <.05, heterogeneity was considered present, and the random-effects model was used. Otherwise, the fixed-effects model was used. Subgroup analyses were conducted to explore the sources of heterogeneity. The sensitivity analysis based on the leave-one-out method was performed to verify the robustness of the results. Publication bias was assessed using Egger tests and by visually examining the symmetry of funnel plots. If the *P* value from the Egger tests <.05 or the funnel plots show significant asymmetry, publication bias was considered present, and the trim-and-fill method was then used [79].

### Assessment of Bias Risk and Certainty of Evidence

Two researchers (HZ and Jingyi Zhou, MEd) independently assessed the risk of bias for included trials. For controlled trials, Cochrane Risk of Bias tool 2.0 was used [80], while the Methodological Index for Non-Randomized Studies tool was used for prepost trials [81]. For detailed bias risk assessment information, refer to Appendix S2 to S3 (P1 - 2) in Multimedia Appendix 2.

The certainty of evidence for the 2 primary outcomes was assessed using the GRADE (Grading of Recommendations Assessment, Development and Evaluation) approach [82]. GRADE starts by assuming high evidence quality, which researchers then evaluate for potential downgrading based on 5 domains: study limitations, inconsistency of results, indirectness of evidence, imprecision, and publication bias. The final certainty of evidence is categorized as high, moderate, low, or very low. For detailed information on evidence certainty assessment, refer to Appendix S4 (P1-2) in Multimedia Appendix 3.

This study was registered in PROSPERO (CRD42025639976) and follows the PRISMA (Preferred Reporting Items for Systematic Reviews and Meta-Analyses) guidelines for reporting [83]. The PRISMA checklist has been uploaded as Checklist 1.

## Results

### Screening Results and Characteristics of Studies

**Figure 1.**
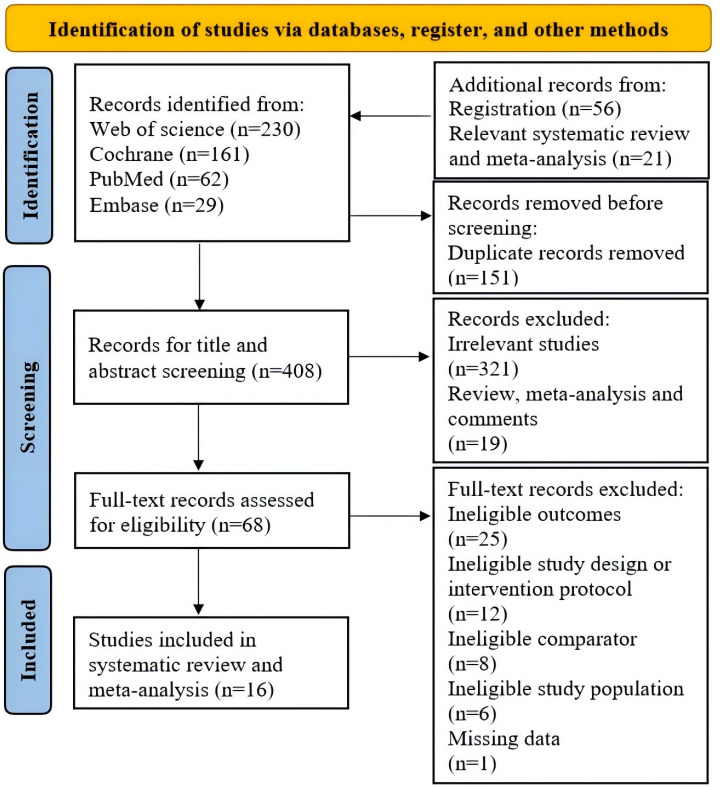
PRISMA flowchart for systematic review and meta-analysis screening process. PRISMA: Preferred Reporting Items for Systematic Reviews and Meta-Analyses.

We retrieved a total of 482 publications from 4 databases and identified 56 records from clinical trial registration and 21 records from the reference lists of related systematic reviews and meta-analyses. After removing duplicates and conducting initial screening, 68 studies were selected for full-text screening. Ultimately, 16 studies comprising 17 trials were included in this systematic review and meta-analysis. Figure 1 outlines the detailed screening procedure, including the number of studies excluded at each stage and the corresponding reasons for exclusion.

The characteristics of the included trials were summarized in Table 2. These trials involved 503 participants aged 6 to 25 years, of whom 322 were children younger than 18 years (from 11 trials), and 181 were young adults older than 18 years (from 6 trials). In the 11 trials with children, the average age of participants in 9 of the 11 trials was around 10 years or older, while the remaining 2 trials reported average ages of 8.25 (SD 1.5) and 7.53 (SD 0.5) years. In the 6 trials involving young adults, the average age of participants in 5 trials was approximately 23 years, with the remaining trial reporting an average age of 20.36 (SD 1.57) years. Among the 322 children, 196 were overweight or obese, while 126 had a normal BMI. In contrast, among the young adults, 29 were overweight or obese, while 152 had a normal BMI. Most trials were conducted in laboratory settings (14 trials), with 3 pediatric trials implemented in nonlaboratory settings (eg, at home). Of the 17 trials, 11 were medium to long-term AVGs with a mean duration of 10.09 (SD 6.99) weeks, spanning from 4 weeks to 30 weeks, while the remaining trials were single-session AVGs. The risk of bias assessment indicated that 2 controlled studies were high risk, 6 raised some concerns, and 2 were low risk, while the prepost studies had a mean Methodological Index for Non-Randomized Studies score of 8.43 (SD 1.4; Appendix S2 to S3, P1 to 2 in Multimedia Appendix 2).

**Table 2.** Characteristics of the included 17 trials in this systematic review and meta-analysis.

Basic information			Participants characteristics			Intervention characteristics			
Author, year	Country or region	Study design	Sample size (sex)	Age range, mean (SD)	BMI category, mean (SD or percentile)	Conditions	Duration	Frequency	Time (each time)
Barbosa et al [21], 2021	Brazil	Prepost trial	14 (5 female and 9 male)	13.3 (2.1)	Normal, 21.48 (4.22)	Laboratory	8 weeks	3 times per week	50 minutes
Bethea et al [42], 2012	America	Prepost trial	28 (10 female and 18 male)	9.9 (0.7)	Normal, 19.8 (3.9)	Nonlaboratory	30 weeks	3 days per week at school, without restriction at home	30 minutes
van Biljon et al [41], 2021	South Africa	Parallel controlled trial	31 (not reported)	11.40 (0.86)	Overweight or obesity (more than the 85th percentile)	Laboratory	6 weeks	3 times per week	30 minutes
Carrasco et al [43], 2013	Brazil	Prepost trial	4 (4 male)	8.25 (1.5)	Overweight or obesity, 23.01 (1.9)	Laboratory	3 weeks	3 times per week	60 minutes
Maloney et al [45], 2008	America	Parallel controlled trial	60 (30 female and 30 male)	7.53 (0.5)	Normal, 17.47 (2.73)	Nonlaboratory	10 weeks	Encouraged 4 times per week	Not applicable
Murphy et al [29], 2009	America	Parallel controlled trial	35 (17 female and 18 male)	10.21 (1.6)	Overweight or obesity (more than the 85th percentile)	Nonlaboratory	12 weeks	Encouraged 5 days per week	Not applicable
Ramos et al [30], 2023	Brazil	Parallel controlled trial	61 (33 female and 28 male)	10 to 16	Overweight or obesity (not reported)	Laboratory	8 weeks	3 times per week	50 minutes
Rauber et al [36], 2013	Brazil	Crossover-controlled trial	8 (not reported)	9.8 (0.5)	Normal, 17.4 (4.7)	Laboratory	1 visit	once	30 minutes
Rauber et al [39], 2014	Brazil	Crossover-controlled trial	16 (8 female and 8 male)	9.3 (0.5)	Normal, 18.4 (3.7)	Laboratory	1 visit	once	30 minutes
Staiano et al [35], 2017	America	Parallel controlled trial	41 (41 female)	15.6 (1.3)	Overweight or obesity (more than the 85th percentile)	Laboratory	12 weeks	3 times per week	60 minutes
Park et al [28], 2015	South Korea	Parallel controlled trial	24 (8 female and 16 male)	15 (0.4)	Overweight or obesity, 25 (4.6)	Laboratory	1 visit	once	60 minutes
de Brito-Gomes et al (a) [38], 2019	Brazil	Prepost trial	8 (not reported)	23 (6)	Overweight or obesity, 23.7 (1.9)	Laboratory	1 visit	once	30 minutes
de Brito-Gomes et al (b) [38], 2018	Brazil	Prepost trial	8 (not reported)	23 (6)	Overweight or obesity, 23.7 (1.9)	Laboratory	4 weeks	2 times per week	30 minutes
de Brito-Gomes et al [37], July 2019	Brazil	Prepost trial	14 (7 female and 7 male)	23 (5)	Normal, 22.85 (1.16)	Laboratory	1 visit	once	30 minutes
de Brito-Gomes et al [31], 2021	Brazil	Crossover-controlled trial	10 (3 female and 7 male)	24.9 (7.5)	Normal, 21.5 (2)	Laboratory	1 visit	once	30 minutes
Huang et al [40], 2017	Taiwan, China	Parallel controlled trial	117 (67 female and 50 male)	22.67 (2.05)	Normal, 22.2 (not reported)	Laboratory	12 weeks	3 times per week	30 minutes
Roopchand-Martin et al [44], 2015	Jamaica	Prepost trial	24 (24 female)	20.36 (1.57)	Normal and overweight (normal: 20.66 [1.77] and overweight: 30.51 [5.18])	Laboratory	6 weeks	First 2 weeks: 5 times per week, third and fourth weeks: 4 times per week, and last 2 weeks: 3 times per week	First 2 weeks: 30 minutes; third and fourth weeks: 45 minutes, and last 2 weeks: 60 minutes

### Meta-Analysis and Subgroup Analyses Results in Children and Young Adults (6-25 Years)

**Figure 2.**
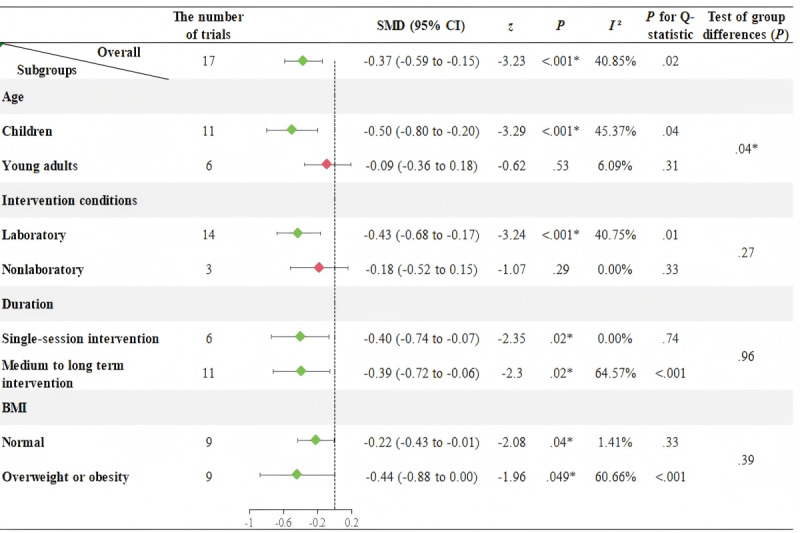
Meta-analysis and subgroup effects of active video games on systolic blood pressure in children and young adults (6–25 years). Subgroup analyses were stratified by age (<18 years [children] vs. ≥18 years [young adults]), intervention setting (laboratory vs. nonlaboratory), duration (single session vs. medium to long term), and body mass index (BMI; normal vs. overweigh or obesity). Diamonds represent subgroup mean effect sizes (indicated by Standardized Mean Difference [SMD]), with horizontal bars indicating 95% confidence intervals (CIs); the vertical dashed line denotes the null effect (SMD=0). Green diamonds indicate statistically significant effects (*P*<.05), while red diamonds denote non-significant results (*P*≥.05). Additionally, because the study by Roopchand-Martin et al [44] includes both participants with normal BMI and those with overweight or obesity, and the authors reported effect sizes for these two subgroups separately, the number of trials in the BMI subgroups is 9 vs. 9.

Figures2  3, respectively, illustrate the effects of AVGs on SBP and DBP in participants aged 6 to 25 years, as well as subgroup analyses results based on age groups, intervention conditions, intervention durations, and BMI.

Figure 2 shows significant heterogeneity among the 17 trials assessing AVGs’ effect on SBP (*I*²=40.85%, *P*=.02). The results from the random-effects model indicate that AVGs can significantly reduce SBP in participants aged 6 to 25 (SMD=−0.37, 95% CI −0.59 to −0.15, *P*<.001). Furthermore, subgroup analyses show that: (1) AVGs significantly reduce SBP in children younger than 18 years (SMD=−0.50, 95% CI −0.80 to −0.20, *P*<.001), but have no significant effect on SBP in young adults older than 18 years (SMD=−0.09, 95% CI−0.36 to 0.18, *P*=.53), with a significant difference between the 2 age groups (*P*=.04); (2) AVGs in laboratory settings significantly reduce SBP (SMD=−0.43, 95% CI−0.68 to −0.17, *P*<.001), while no significant effect is observed in nonlaboratory settings (SMD=−0.18, 95% CI −0.52 to 0.15, *P*=.29), but there is no significant difference between the groups (*P*=.27); (3) both single-session and medium to long-term AVGs significantly reduce SBP (single session: SMD=−0.40, 95% CI −0.74 to −0.07, *P*=.02; medium to long-term interventions: SMD=−0.39, 95% CI −0.72 to −0.06, *P*=.02); and (4) AVGs demonstrate significant effects in reducing SBP among both normal weight and overweight or obese populations (normal: SMD=−0.22, 95% CI −0.43 to −0.01, *P*=.04; overweight or obesity: SMD=−0.44, 95% CI −0.88 to 0.00, *P*=.05).

**Figure 3.**
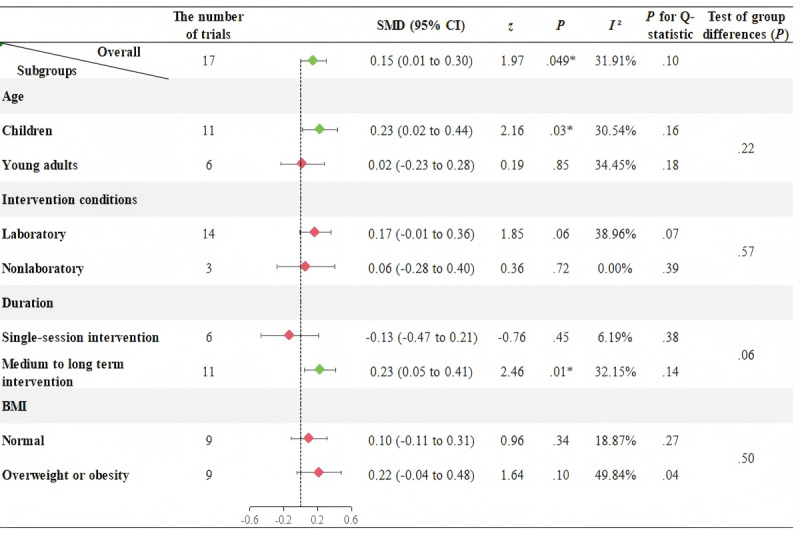
Meta-analysis and subgroup effects of active video games on diastolic blood pressure in children and young adults (6–25 years). Subgroup analyses were stratified by age (<18 years [children] vs. ≥18 years [young adults]), intervention setting (laboratory vs. nonlaboratory), duration (single session vs. medium to long term), and body mass index (BMI; normal vs. overweight or obesity). Diamonds represent subgroup mean effect sizes (indicated by Standardized Mean Difference [SMD]), with horizontal bars indicating 95% confidence intervals (CIs); the vertical dashed line denotes the null effect (SMD=0). Green diamonds indicate statistically significant effects (*P*<.05), while red diamonds denote non-significant results (*P*≥.05). Additionally, because the study by Roopchand-Martin et al [44] includes both participants with normal BMI and those with overweight or obesity, and the authors reported effect sizes for these two subgroups separately, the number of trials in the BMI subgroups is 9 vs. 9.

Figure 3 shows no significant heterogeneity among the 17 trials evaluating the effect of AVGs on DBP (*I*²=31.91%, *P*=.10). The results from the fixed-effects model indicate that AVGs significantly increase DBP in participants aged 6 to 25 years (SMD=0.15, 95% CI 0.01 to 0.30, *P*=.049). Subgroup analyses further reveal that: (1) AVGs can significantly increase DBP in children younger than 18 years (SMD=0.23, 95% CI 0.02 to 0.44, *P*=.03), while no significant effect is found for young adults aged 18 year sand older (SMD=0.02, 95% CI −0.23 to 0.28, *P*=.85); and (2) medium to long term AVGs can exert a significant effect in increasing DBP (SMD=0.23, 95% CI 0.05 to 0.41, *P*=.01).

### Meta-Analysis and Subgroup Analyses Results in Children (<18 Years)

The aforementioned subgroup analyses show that AVGs have more pronounced effects on SBP and DBP in children younger than 18 years (SBP, SMD:−0.50, *P*<.001; DBP, SMD: 0.23, *P*=.03) compared to young adults older than 18 years (SBP, SMD:−0.09, *P*=.53; DBP, SMD: 0.02, *P*=.85). We therefore conduct further analyses on 11 trials only involving children.

**Figure 4.**
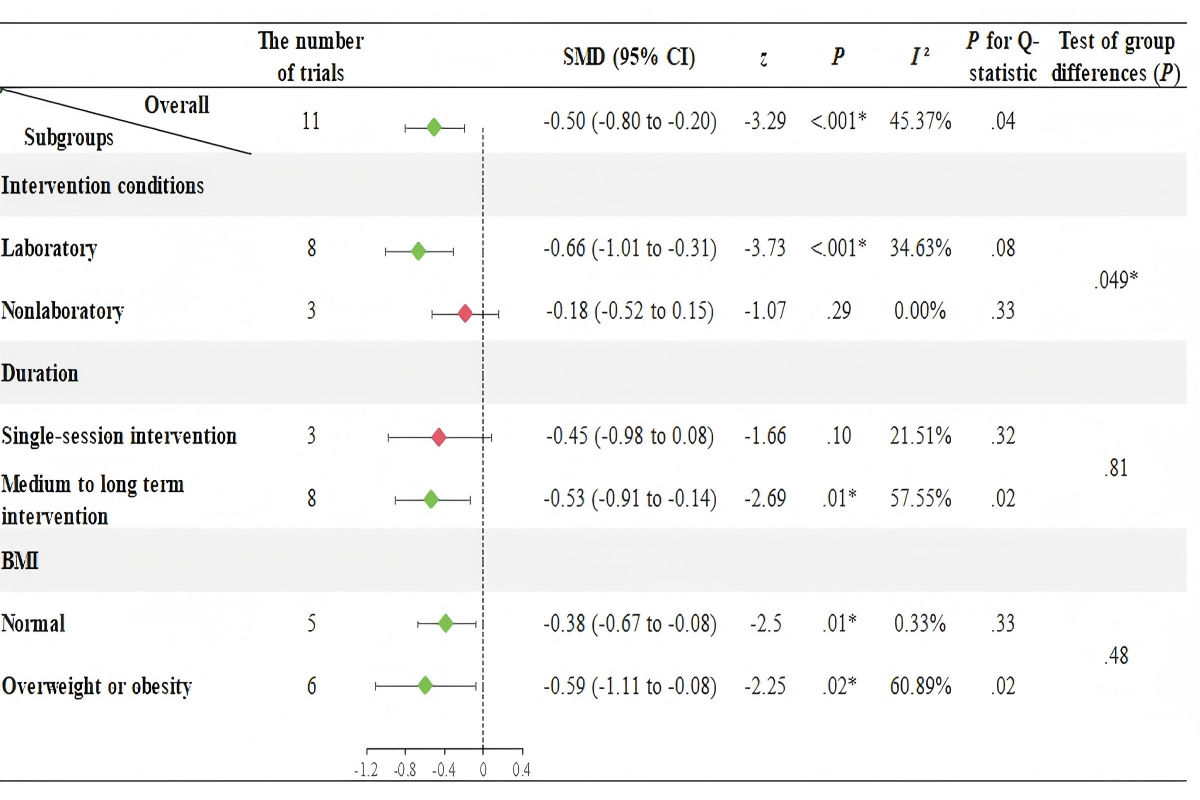
Meta-analysis and subgroup effects of active video games on systolic blood pressure in children <18 years. Subgroup analyses were stratified by intervention setting (laboratory vs. nonlaboratory), duration (single session vs. medium to long term), and body mass index (BMI; normal vs. overweight or obesity). Diamonds represent subgroup mean effect sizes (indicated by Standardized Mean Difference [SMD]), with horizontal bars indicating 95% confidence intervals (CIs); the vertical dashed line denotes the null effect (SMD=0). Green diamonds indicate statistically significant effects (*P*<.05), while red diamonds denote non-significant results (*P*≥.05).

**Figure 5.**
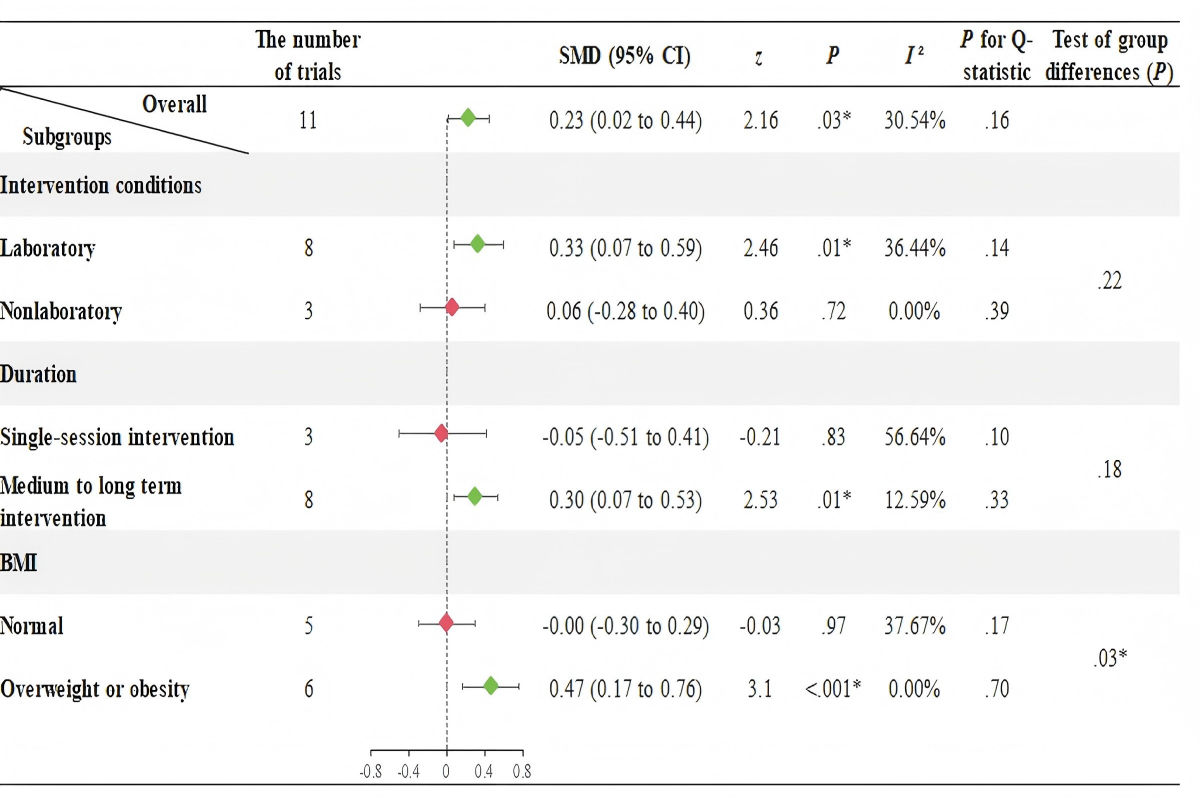
Meta-analysis and subgroup effects of active video games on diastolic blood pressure in children <18 years. Subgroup analyses were stratified by intervention setting (laboratory vs. nonlaboratory), duration (single session vs. medium to long term), and body mass index (BMI; normal vs. overweight or obesity). Diamonds represent subgroup mean effect sizes (indicated by Standardized Mean Difference [SMD]), with horizontal bars indicating 95% confidence intervals (CIs); the vertical dashed line denotes the null effect (SMD=0). Green diamonds indicate statistically significant effects (*P*<.05), while red diamonds denote non-significant results (*P*≥.05).

As observed in Figure 4, significant heterogeneity was found in the effects on SBP across the 11 trials (*I*²=45.37%, *P*=.04), thus the random-effects model is used. In contrast, Figure 5 shows no significant heterogeneity in the effects on DBP across the 11 trials (*I*²=30.54%, *P*=.16), so the fixed-effects model is used.

Figure 4 and Figure 5 indicate that AVGs in laboratory settings significantly reduce SBP (SMD=−0.66, 95% CI −1.01 to −0.31, *P*<.001) and increase DBP (SMD=0.33, 95% CI 0.07 to 0.59, *P*=.01) in children younger than 18 years. However, AVGs outside laboratory settings do not induce reductions in SBP or increases in DBP. Additionally, medium to long-term AVGs were found to significantly reduce SBP (SMD=−0.53, 95% CI −0.91 to −0.14, *P*=.01) and increase DBP (SMD=0.30, 95% CI 0.07 to 0.53, *P*=.01) in children, whereas single-session AVGs did not produce significant changes in SBP and DBP. In overweight or obese children, we found that AVGs could reduce SBP (SMD=−0.59, 95% CI −1.11 to −0.08, *P*=.02) and increase DBP (SMD=0.47, 95% CI 0.17 to 0.76, *P*<.001). In contrast, among children with normal BMI, AVGs only significantly reduce SBP (SMD=−0.38, 95% CI −0.67 to −0.08, *P*=.01), with no significant changes in DBP (SMD=−0.001, 95% CI −0.30 to 0.29, *P*=.97).

### Results of Sensitivity Analyses, Egger Tests, and GRADE Assessments

This study performs 3 sensitivity analyses to validate the robustness of the results. First, we reanalyzed all controlled trials after excluding 7 prepost trials, revealing that AVGs significantly reduce SBP and increase DBP (Figure S1 to Figure S2 in Multimedia Appendix 4). Second, after excluding 3 trials with BP measurements taken in nonlaboratory settings, the effect sizes of the remaining trials are repooled. The results still indicate that the effect of AVGs on reducing SBP and increasing DBP remains significant (Figure S3 to Figure S4 in Multimedia Appendix 4). Lastly, sensitivity analyses based on the leave-one-out method are also used in this study. The results demonstrate that AVGs have a stable effect in reducing SBP (Table 3). However, the effects of AVGs on increasing DBP might be influenced by a single trial, leading to nonsignificant outcomes (Table 3).

Furthermore, results of Egger tests and the funnel plots indicate publication bias in the effect of AVGs on the SBP of participants aged 6 to 25 years (Table 3 and Appendix S5 in Multimedia Appendix 5). After applying the trim-and-fill method, the effect size changes from −0.37 (95% CI −0.59 to −0.15) to −0.19 (95% CI −0.47 to 0.10; Appendix S6 in Multimedia Appendix 6), suggesting that further studies are required to consolidate the evidence. Based on the information, the GRADE assessments indicate that the certainties of evidence for SBP in participants aged 6 to 25 years and children younger than 18 years were rated as low, while those for DBP were rated as moderate (Appendix S4, P1 - 2 in Multimedia Appendix 3).

**Table 3.** Results of sensitivity analyses and Egger tests for blood pressure outcomes.

Populations and outcomes	Effect sizes (*P* value)	Sensitivity analyses		Publication bias
		SMD^a^ (range)	*P* value (range)	*P* value for Egger tests
Children and young adults (6 to 25 years)				
SBP^b^	−0.37 (<.001)	−0.42 to −0.24	<.001 to .006	.03^d^
DBP^c^	0.15 (.049)	0.12 to 0.18^d^	.03 to .21^d^	.08
Children (<18 years)				
SBP	−0.50 (<.001)	−0.58 to −0.35	<.001 to .004	.22
DBP	0.23 (.03)	0.17 to 0.31^d^	.007 to .13^d^	.86

a SMD: standardized mean difference

b SBP: systolic blood pressure

c DBP: diastolic blood pressure

d Indicates failure to pass sensitivity analysis or Egger test.

## Discussion

### Principal Results

To our knowledge, this is the first systematic review and meta-analysis to evaluate the effects of AVGs on BP. The findings demonstrate that AVGs significantly reduce SBP and increase DBP in children younger than 18 years.

First, the finding that AVGs can help reduce SBP is notable, particularly given that even a marginal elevation of 1 to 2 mm Hg in SBP may significantly increase the risk of CVDs [84-86]. SBP levels during youth are considered important predictors of hypertension risk in adulthood [3]. Our findings support previous studies suggesting that AVGs can help regulate SBP [21,30,31,35-38]. Additionally, DBP, another critical indicator of cardiovascular health, demonstrates a significant increase following AVGs intervention, contrasting with nonsignificant trends observed in previous studies [30,39,40,42]. AVGs can simultaneously reduce SBP while increasing DBP in participants due to distinct physiological mechanisms. Slow-paced, relaxing games lower SBP by reducing the sympathetic tone and cardiac output [87,88], while rhythm games may further decrease SBP through controlled breathing and parasympathetic activation [89,90]. Conversely, prolonged standing and mental stress during gaming trigger peripheral vasoconstriction (via adrenaline or noradrenaline release), elevating vascular resistance and DBP [91]. Additionally, isometric muscle tension from gripping controllers tightly can compress blood vessels, further increasing DBP [92]. This divergence highlights how physical effects differentially influence SBP (cardiac-driven) and DBP (vascular resistance-driven). The combined effect of reduced SBP and increased DBP can lead to decreased pulse pressure (PP=SBP–DBP) while partially offsetting SBP-driven reductions in mean arterial pressure (mean arterial pressure=1/3 SBP +2/3 DBP). This indicates that AVGs may improve vascular elasticity while maintaining stable organ perfusion pressure. Reduced PP can prevent excessive arterial stretch, delay arterial fatigue, and avoid the fracture of elastic elements, reducing the risk of intimal injury that leads to atherosclerosis and thrombosis [93]. Meanwhile, stable mean arterial pressure can help preserve normal organ perfusion, tissue oxygenation, and circulatory homeostasis [94].

However, further subgroup analysis demonstrated that these promising effects of AVGs were only found in children younger than 18 years and not in young adults older than 18 years. The discrepancy may be attributed to age-related differences in endothelial function. As Holder et al [95] reported, systemic endothelial function—measured by flow-mediated dilation—inevitably declines with age. The higher endothelial function in children is an important factor against CVDs and may also explain their heightened susceptibility to BP control through AVGs [96]. Endothelial function in children may help manage BP in ways lost to adults, indicating that AVGs targeted at a younger age may have more beneficial effects on cardiovascular health. As suggested by the American Heart Association [97], integrating AVGs into lifestyle routines during the critical developmental period may provide favorable conditions for the development and improvement of the cardiovascular system.

Among the included trials, 3 carried out AVGs under nonlaboratory conditions. Despite participants showing 90% adherence in these trials [45], the results indicated no significant effects of AVGs on SBP and DBP in a nonlaboratory setting [29,42,45]. The significant disparity in BP responses between laboratory and nonlaboratory settings may be attributed to variations in intervention fidelity and physiological stressor intensity. Structured laboratory environments ensure consistent exercise duration and intensity, which is difficult to achieve in nonlaboratory settings. In such an unregulated setting, children may struggle to sustain attention during prolonged AVGs participation and may not engage in them regularly and systematically to achieve the “threshold dose” [97,98]. Moreover, the absence of initial movement skills instruction and goal-setting among home-based AVG users may further diminish their effectiveness [99,100]. To translate the BP benefits of AVGs observed in laboratory conditions into broader practice, future AVGs should consider integrating digital strategies (such as streak counters, weekly goals, and rewards such as unlocking new levels for completion rate) and wearable devices (such as heart rate and calorie monitors) into the systems.

From the perspective of intervention duration, medium to long-term AVGs demonstrated significant effects in reducing SBP and increasing DBP. In fact, most lifestyle interventions need a long time to induce beneficial changes [101]. The human body is a highly complex and sophisticated system where physiological functions interact dynamically to maintain balance [102]. When health interventions are applied to it, they do not merely affect a single indicator but require multiple systems to gradually adapt and adjust [103]. In the cardiovascular system, BP regulation involves the coordinated functioning of the heart, blood vessels, and neuroendocrine systems [11]. AVGs may require time to interact with these physiological mechanisms, shifting the original balance to a new and healthier equilibrium. Moreover, the new equilibrium needs to be maintained to achieve the expected health benefits [104], which highlights the importance of integrating interventions into daily life. Given that playing video games is a common modern pastime, cultivating habits of sustained participation in engaging AVGs may be both feasible and promising [97].

Furthermore, greater changes in SBP and DBP were observed in overweight or obese participants. This finding may be attributed to the amelioration of endothelial dysfunction typically present in this population [28,31] following engagement with AVGs. Previous studies have supported a significant correlation between obesity and endothelial dysfunction [105]. Endothelial cells play a critical role in BP regulation by dynamically modulating vascular tone through the secretion of vasoactive substances such as nitric oxide, prostacyclin, and endothelium-derived hyperpolarizing factors [106]. Participation in AVGs has been shown to enhance nitric oxide bioavailability and augment brachial artery flow-mediated dilation, thereby mitigating endothelial dysfunction [28], which may contribute to the greater BP effects observed in overweight or obese participants. In addition to the aforementioned mechanism, the increase in physical activity induced by AVGs is believed to enhance insulin sensitivity and reduce insulin resistance in overweight and obese populations [107,108]. Improved insulin sensitivity may benefit BP management in this population by regulating sympathetic nervous system activity and reducing renal sodium retention [109,110].

### Limitations

The findings of this study need to be interpreted with the following limitations. First, the participants included in this study had an age range of 6 to 25 years, which may introduce heterogeneity. Future studies should adopt narrower age bands or developmental stages to reduce confounding. Second, GRADE assessments indicate low certainty of evidence for SBP changes. The heterogeneity across trials and limited sample sizes suggests that further research is needed to solidify the evidence on the health significance of AVGs. Furthermore, the inclusion of prepost trials in this study may limit the causal inferences regarding AVGs’ specific effect on BP. Prepost studies, by lacking a control group, are unable to rule out confounding variables that may influence BP outcomes. Although the reanalysis of the controlled trials confirmed the robustness of the results, caution is still needed when interpreting them. Future research using randomized controlled trials with parallel control groups will be critical to strengthen causal claims about AVGs’ effects on BP. Lastly, many studies did not report exercise intensity in detail, limiting our ability to explore the optimal exercise prescription of AVGs for managing BP. Future studies are encouraged to develop and report AVGs protocols following the FITT (frequency, intensity, time, and type) principle to derive more detailed dose-response relationships and strengthen the translational value of AVGs as an evidence-based intervention.

### Conclusion

Our research suggests that AVGs may serve as a promising strategy for pediatric BP management, demonstrating significant effects in reducing SBP and increasing DBP. More high-quality trials are called for to validate their cardiovascular benefits and clinical value.

## Supplementary material

10.2196/75000 Multimedia Appendix 1 Search strategies for different sources.

10.2196/75000 Multimedia Appendix 2 Cochrane Risk of Bias tool 2.0 for the included controlled trials.

10.2196/75000 Multimedia Appendix 3 Details of evidence certainty assessment using GRADE. GRADE: Grading of Recommendations Assessment, Development and Evaluation.

10.2196/75000 Multimedia Appendix 4 Sensitivity analysis results from controlled trials and those from trials with blood pressure measurements taken in laboratory settings.

10.2196/75000 Multimedia Appendix 5 Funnel plots for publication bias.

10.2196/75000 Multimedia Appendix 6 The funnel plot and effect size after using the trim and fill method.

10.2196/75000 Checklist 1 The PRISMA checklists for the main text and abstract.

## Abbreviations

AVG: active video game
BP: blood pressure
CVD: cardiovascular disease
DBP: diastolic blood pressure
FITT: frequency, intensity, time, and type
GRADE: Grading of Recommendations Assessment, Development, and Evaluations
PICOS: Participants, Intervention, Comparison, Outcome, and Study Design
PP: pulse pressure
PRISMA: Preferred Reporting Items for Systematic Reviews and Meta-Analyses
SBP: systolic blood pressure
SMD: standardized mean difference
