# Supplementary material for: Role of Active Video Games in Blood Pressure Management Among Children and Young Adults: Systematic Review and Meta-Analysis
Source: J Med Internet Res. 2025 Aug 19;27:e75000. doi: 10.2196/75000 (PMC12381676; doi:10.2196/75000)
Supplement: Multimedia Appendix 1 [file jmir-v27-e75000-s001.docx]

**Appendix S1.** Search Strategies

**Web of science** (n=230)

1. TS=(Blood Pressure OR Hypotension OR Hypertension OR Pressure, Blood OR Pulse Pressure OR Pressure, Pulse OR Diastolic Pressure OR Pressure, Diastolic OR Systolic Pressure OR Pressures, Systolic OR Pressure, Systolic OR Blood Pressure Determinations OR Determination, Blood Pressure OR Arterial Pressures OR Pressure, Arterial OR Pressures, Arterial OR Arterial Blood Pressure OR Arterial Blood Pressures OR Blood Pressures, Arterial OR Pressure, Arterial Blood OR Pressures, Arterial Blood OR Arterial Tension OR Arterial Tensions OR Tension, Arterial OR Tensions, Arterial OR Blood Pressure, Arterial OR Mean Arterial Pressure OR Arterial Pressure, Mean OR Arterial Pressures, Mean OR Mean Arterial Pressures OR Pressure, Mean Arterial OR Pressures, Mean Arterial OR Aortic Pressure OR Aortic Pressures OR Pressure, Aortic OR Pressures, Aortic OR Aortic Blood Pressure OR Aortic Blood Pressures OR Blood Pressures, Aortic OR Pressure, Aortic Blood OR Pressures, Aortic Blood OR Aortic Tension OR Aortic Tensions OR Tension, Aortic OR Tensions, Aortic OR Blood Pressure, Aortic OR Aortic Pulse Pressure OR Aortic Pulse Pressures OR Pressure, Aortic Pulse OR Pressures, Aortic Pulse OR Pulse Pressure, Aortic OR Pulse Pressures, Aortic OR Mean Aortic Pressure OR Aortic Pressure, Mean OR Aortic Pressures, Mean OR Mean Aortic Pressures OR Pressure, Mean Aortic OR Pressures, Mean Aortic OR Ambulatory Blood Pressure Monitoring OR Monitoring, Ambulatory Blood Pressure OR Blood Pressure Monitoring, Home OR Home Blood Pressure Monitoring OR Blood Pressure Monitoring, Self OR Self Blood Pressure Monitoring OR Pressures, Venous OR Pressure, Venous OR Venous Pressures OR Blood Pressure, Venous OR Blood Pressures, Venous OR Pressures, Venous Blood OR Pressure, Venous Blood OR Venous Blood Pressure OR Venous Blood Pressures OR Blood Pressure, Low OR Hypotension, Vascular OR Low Blood Pressure OR Vascular Hypotension OR Blood Pressure, High OR Blood Pressures, High OR High Blood Pressure OR High Blood Pressures OR Blood Pressure Change)

2. TS=(Exergame OR Exergames OR Exergaming OR Exergamings OR Active Video Game OR Active Video Games OR Virtual Reality Exercise OR Exercises, Virtual Reality OR Exercise, Virtual Reality OR Virtual Reality Exercises OR Active-Video Gaming OR Active Video Gaming OR Active-Video Gamings OR Gaming, Active-Video OR Gamings, Active-Video OR Exergames OR interactive video game OR Somatosensory Games OR Motion-controlled video games OR Physical activity video games OR Movement-based video games OR Fitness video games OR active screen time game OR Dance Revolution OR gamercize power stepper OR Wii FitTM OR Wii OR Nintendo Wii OR Kinect OR interactive bicycle games OR dance videogame OR Gesture-based Game OR Ring Fit Adventure OR Super Mario Party OR Fitness Boxing OR Just Dance OR AR Gaming OR VR Fitness OR Motion-Sensing Game)

3. TS=(Child OR Adolescent OR young adults OR Children OR Adolescents OR Adolescence OR Adolescents, Female OR Adolescent, Female OR Female Adolescent OR Female Adolescents OR Adolescents, Male OR Adolescent, Male OR Male Adolescent OR Male Adolescents OR Youth OR Youths OR Teens OR Teen OR Teenagers OR Teenager OR Adults, Young OR Adult, Young OR Young Adults)

1 AND 2 AND 3

**Cochrane library** (n=161)

1. MeSH descriptor: [Blood Pressure] explode all trees OR 'Blood Pressure' OR 'Hypotension' OR 'Hypertension' OR 'Pressure, Blood' OR 'Pulse Pressure' OR 'Pressure, Pulse' OR 'Diastolic Pressure' OR 'Pressure, Diastolic' OR 'Systolic Pressure' OR 'Pressures, Systolic' OR 'Pressure, Systolic' OR 'Blood Pressure Determinations' OR 'Determination, Blood Pressure' OR 'Arterial Pressures' OR 'Pressure, Arterial' OR 'Pressures, Arterial' OR 'Arterial Blood Pressure' OR 'Arterial Blood Pressures' OR 'Blood Pressures, Arterial' OR 'Pressure, Arterial Blood' OR 'Pressures, Arterial Blood' OR 'Arterial Tension' OR 'Arterial Tensions' OR 'Tension, Arterial' OR 'Tensions, Arterial' OR 'Blood Pressure, Arterial' OR 'Mean Arterial Pressure' OR 'Arterial Pressure, Mean' OR 'Arterial Pressures, Mean' OR 'Mean Arterial Pressures' OR 'Pressure, Mean Arterial' OR 'Pressures, Mean Arterial' OR 'Aortic Pressure' OR 'Aortic Pressures' OR 'Pressure, Aortic' OR 'Pressures, Aortic' OR 'Aortic Blood Pressure' OR 'Aortic Blood Pressures' OR 'Blood Pressures, Aortic' OR 'Pressure, Aortic Blood' OR 'Pressures, Aortic Blood' OR 'Aortic Tension' OR 'Aortic Tensions' OR 'Tension, Aortic' OR 'Tensions, Aortic' OR 'Blood Pressure, Aortic' OR 'Aortic Pulse Pressure' OR 'Aortic Pulse Pressures' OR 'Pressure, Aortic Pulse' OR 'Pressures, Aortic Pulse' OR 'Pulse Pressure, Aortic' OR 'Pulse Pressures, Aortic' OR 'Mean Aortic Pressure' OR 'Aortic Pressure, Mean' OR 'Aortic Pressures, Mean' OR 'Mean Aortic Pressures' OR 'Pressure, Mean Aortic' OR 'Pressures, Mean Aortic' OR 'Ambulatory Blood Pressure Monitoring' OR 'Monitoring, Ambulatory Blood Pressure' OR 'Blood Pressure Monitoring, Home' OR 'Home Blood Pressure Monitoring' OR 'Blood Pressure Monitoring, Self' OR 'Self Blood Pressure Monitoring' OR 'Pressures, Venous' OR 'Pressure, Venous' OR 'Venous Pressures' OR 'Blood Pressure, Venous' OR 'Blood Pressures, Venous' OR 'Pressures, Venous Blood' OR 'Pressure, Venous Blood' OR 'Venous Blood Pressure' OR 'Venous Blood Pressures' OR 'Blood Pressure, Low' OR 'Hypotension, Vascular' OR 'Low Blood Pressure' OR 'Vascular Hypotension' OR 'Blood Pressure, High' OR 'Blood Pressures, High' OR 'High Blood Pressure' OR 'High Blood Pressures' OR 'Blood Pressure Change'

2. MeSH descriptor: [Exergaming] explode all trees OR 'Exergame' OR 'Exergames' OR 'Exergaming' OR 'Exergamings' OR 'Active Video Game' OR 'Active Video Games' OR 'Virtual Reality Exercise' OR 'Exercises, Virtual Reality' OR 'Exercise, Virtual Reality' OR 'Virtual Reality Exercises' OR 'Active-Video Gaming' OR 'Active Video Gaming' OR 'Active-Video Gamings' OR 'Gaming, Active-Video' OR 'Gamings, Active-Video' OR 'Exergames' OR 'Interactive Video Game' OR 'Somatosensory Games' OR 'Motion-controlled Video Games' OR 'Physical Activity Video Games' OR 'Movement-based Video Games' OR 'Fitness Video Games' OR 'Active Screen Time Game' OR 'Dance Revolution' OR 'Gamercize Power Stepper' OR 'Wii FitTM' OR 'Wii' OR 'Nintendo Wii' OR 'Kinect' OR 'Interactive Bicycle Games' OR 'Dance Videogame' OR 'Gesture-based Game' OR 'Ring Fit Adventure' OR 'Super Mario Party' OR 'Fitness Boxing' OR 'Just Dance' OR 'AR Gaming' OR 'VR Fitness' OR 'Motion-Sensing Game'

3. MeSH descriptor: [Child] explode all trees OR 'Child' OR 'Adolescent' OR 'young adults' OR 'Children' OR 'Adolescents' OR 'Adolescence' OR 'Adolescents, Female' OR 'Adolescent, Female' OR 'Female Adolescent' OR 'Female Adolescents' OR 'Adolescents, Male' OR 'Adolescent, Male' OR 'Male Adolescent' OR 'Male Adolescents' OR 'Youth' OR 'Youths' OR 'Teens' OR 'Teen' OR 'Teenagers' OR 'Teenager' OR 'Adults, Young' OR 'Adult, Young' OR 'Young Adults'

1 AND 2 AND 3

**PubMed** (n=62)

1. "Blood Pressure"[Mesh] OR "Hypotension"[Mesh] OR "Hypertension"[Mesh] OR "Pressure, Blood" OR "Pulse Pressure" OR "Pressure, Pulse" OR "Diastolic Pressure" OR "Pressure, Diastolic" OR "Systolic Pressure" OR "Pressures, Systolic" OR "Pressure, Systolic" OR "Blood Pressure Determinations" OR "Determination, Blood Pressure" OR "Arterial Pressures" OR "Pressure, Arterial" OR "Pressures, Arterial" OR "Arterial Blood Pressure" OR "Arterial Blood Pressures" OR "Blood Pressures, Arterial" OR "Pressure, Arterial Blood" OR "Pressures, Arterial Blood" OR "Arterial Tension" OR "Arterial Tensions" OR "Tension, Arterial" OR "Tensions, Arterial" OR "Blood Pressure, Arterial" OR "Mean Arterial Pressure" OR "Arterial Pressure, Mean" OR "Arterial Pressures, Mean" OR "Mean Arterial Pressures" OR "Pressure, Mean Arterial" OR "Pressures, Mean Arterial" OR "Aortic Pressure" OR "Aortic Pressures" OR "Pressure, Aortic" OR "Pressures, Aortic" OR "Aortic Blood Pressure" OR "Aortic Blood Pressures" OR "Blood Pressures, Aortic" OR "Pressure, Aortic Blood" OR "Pressures, Aortic Blood" OR "Aortic Tension" OR "Aortic Tensions" OR "Tension, Aortic" OR "Tensions, Aortic" OR "Blood Pressure, Aortic" OR "Aortic Pulse Pressure" OR "Aortic Pulse Pressures" OR "Pressure, Aortic Pulse" OR "Pressures, Aortic Pulse" OR "Pulse Pressure, Aortic" OR "Pulse Pressures, Aortic" OR "Mean Aortic Pressure" OR "Aortic Pressure, Mean" OR "Aortic Pressures, Mean" OR "Mean Aortic Pressures" OR "Pressure, Mean Aortic" OR "Pressures, Mean Aortic" OR "Ambulatory Blood Pressure Monitoring" OR "Monitoring, Ambulatory Blood Pressure" OR "Blood Pressure Monitoring, Home" OR "Home Blood Pressure Monitoring" OR "Blood Pressure Monitoring, Self" OR "Self Blood Pressure Monitoring" OR "Pressures, Venous" OR "Pressure, Venous" OR "Venous Pressures" OR "Blood Pressure, Venous" OR "Blood Pressures, Venous" OR "Pressures, Venous Blood" OR "Pressure, Venous Blood" OR "Venous Blood Pressure" OR "Venous Blood Pressures" OR "Blood Pressure, Low" OR "Hypotension, Vascular" OR "Low Blood Pressure" OR "Vascular Hypotension" OR "Blood Pressure, High" OR "Blood Pressures, High" OR "High Blood Pressure" OR "High Blood Pressures" OR "Blood Pressure Change"

2. "Video Games"[Mesh] OR "Exergaming"[Mesh] OR "Exergame" OR "Exergames" OR "Exergaming" OR "Exergamings" OR "Active Video Game" OR "Active Video Games" OR "Virtual Reality Exercise" OR "Exercises, Virtual Reality" OR "Exercise, Virtual Reality" OR "Virtual Reality Exercises" OR "Active-Video Gaming" OR "Active Video Gaming" OR "Active-Video Gamings" OR "Gaming, Active-Video" OR "Gamings, Active-Video" OR "Exergames" OR "Exergame" OR "interactive video game" OR "Somatosensory Games" OR "Motion-controlled video games" OR "Physical activity video games" OR "Movement-based video games" OR "Fitness video games" OR "active screen time game" OR "Dance Revolution" OR "gamercize power stepper" OR "Wii FitTM" OR "Wii" OR "Nintendo Wii" OR "Kinect" OR "interactive bicycle games" OR "dance videogame" OR "Gesture-based Game" OR "Ring Fit Adventure" OR "Super Mario Party" OR "Fitness Boxing" OR "Just Dance" OR "AR gaming" OR "VR fitness" OR "Motion-sensing game"

3. "Child"[Mesh] OR "Adolescent"[Mesh] OR "Young Adult"[Mesh] OR "Children" OR "Adolescents" OR "Adolescence" OR "Adolescents, Female" OR "Adolescent, Female" OR "Female Adolescent" OR "Female Adolescents" OR "Adolescents, Male" OR "Adolescent, Male" OR "Male Adolescent" OR "Male Adolescents" OR "Youth" OR "Youths" OR "Teens" OR "Teen" OR "Teenagers" OR "Teenager" OR "Adults, Young" OR "Adult, Young" OR "Young Adults"

1 AND 2 AND 3

**Embase** (n=29)

1. blood pressure/ or (Blood Pressure or Increase in blood pressure or Normal blood pressure or Systolic blood pressure or Diastolic blood pressure or Systemic arterial pressure or Blood pressure cuff or Venous Blood Pressure or Labile blood pressure or Blood pressure alteration finding or Segmental pressure blood pressure or Abnormal blood pressure or Orthostatic blood pressure or Blood pressure fluctuation or Standing blood pressure or Lying blood pressure or Blood pressure regulation or Blood pressure ambulatory or Blood pressure problem or Blood pressure change).mp.

2. 'Exergame ' or 'Exergames ' or 'Exergaming' or 'Exergamings' or 'Active Video Game' or 'Active Video Games' or 'Virtual Reality Exercise' or 'Exercises, Virtual Reality' or 'Exercise, Virtual Reality' or 'Virtual Reality Exercises' or 'Active-Video Gaming' or 'Active Video Gaming' or 'Active-Video Gamings' or 'Gaming, Active-Video' or 'Gamings, Active-Video' or 'interactive video game' or 'Somatosensory Games' or 'Motion-controlled video games' or 'Physical activity video games' or 'Movement-based video games' or 'Fitness video games' or 'active screen time game' or 'Dance Revolution' or 'gamercize power stepper' or 'Wii FitTM' or 'Wii' or 'Nintendo Wii' or 'Kinect' or 'interactive bicycle games' or 'dance videogame' or 'Gesture-based Game' or 'Ring Fit Adventure' or 'Super Mario Party' or 'Fitness Boxing' or 'Just Dance' or 'AR Gaming' or 'VR Fitness' or 'Motion-Sensing Game'

3. 'Child' OR 'Adolescent' OR 'young adult' OR 'Children' OR 'Adolescents' OR 'young adults' OR 'Adolescence' OR 'Adolescents, Female' OR 'Adolescent, Female' OR 'Female Adolescent' OR 'Female Adolescents' OR 'Adolescents, Male' OR 'Adolescent, Male' OR 'Male Adolescent' OR 'Male Adolescents' OR 'Youth' OR 'Youths' OR 'Teens' OR 'Teen' OR 'Teenagers' OR 'Teenager' OR 'Adults, Young' OR 'Adult, Young'

1 AND 2 AND 3

**Registration** (ClinicalTrials.gov, 56)

1. "Blood Pressure" OR "Hypotension" OR "Hypertension" OR "Pressure, Blood" OR "Pulse Pressure" OR "Pressure, Pulse" OR "Diastolic Pressure" OR "Pressure, Diastolic" OR "Systolic Pressure" OR "Pressures, Systolic" OR "Pressure, Systolic" OR "Blood Pressure Determinations" OR "Determination, Blood Pressure" OR "Arterial Pressures" OR "Pressure, Arterial" OR "Pressures, Arterial" OR "Arterial Blood Pressure" OR "Arterial Blood Pressures" OR "Blood Pressures, Arterial" OR "Pressure, Arterial Blood" OR "Pressures, Arterial Blood" OR "Arterial Tension" OR "Arterial Tensions" OR "Tension, Arterial" OR "Tensions, Arterial" OR "Blood Pressure, Arterial" OR "Mean Arterial Pressure" OR "Arterial Pressure, Mean" OR "Arterial Pressures, Mean" OR "Mean Arterial Pressures" OR "Pressure, Mean Arterial" OR "Pressures, Mean Arterial" OR "Aortic Pressure" OR "Aortic Pressures" OR "Pressure, Aortic" OR "Pressures, Aortic" OR "Aortic Blood Pressure" OR "Aortic Blood Pressures" OR "Blood Pressures, Aortic" OR "Pressure, Aortic Blood" OR "Pressures, Aortic Blood" OR "Aortic Tension" OR "Aortic Tensions" OR "Tension, Aortic" OR "Tensions, Aortic" OR "Blood Pressure, Aortic" OR "Aortic Pulse Pressure" OR "Aortic Pulse Pressures" OR "Pressure, Aortic Pulse" OR "Pressures, Aortic Pulse" OR "Pulse Pressure, Aortic" OR "Pulse Pressures, Aortic" OR "Mean Aortic Pressure" OR "Aortic Pressure, Mean" OR "Aortic Pressures, Mean" OR "Mean Aortic Pressures" OR "Pressure, Mean Aortic" OR "Pressures, Mean Aortic" OR "Ambulatory Blood Pressure Monitoring" OR "Monitoring, Ambulatory Blood Pressure" OR "Blood Pressure Monitoring, Home" OR "Home Blood Pressure Monitoring" OR "Blood Pressure Monitoring, Self" OR "Self Blood Pressure Monitoring" OR "Pressures, Venous" OR "Pressure, Venous" OR "Venous Pressures" OR "Blood Pressure, Venous" OR "Blood Pressures, Venous" OR "Pressures, Venous Blood" OR "Pressure, Venous Blood" OR "Venous Blood Pressure" OR "Venous Blood Pressures" OR "Blood Pressure, Low" OR "Hypotension, Vascular" OR "Low Blood Pressure" OR "Vascular Hypotension" OR "Blood Pressure, High" OR "Blood Pressures, High" OR "High Blood Pressure" OR "High Blood Pressures" OR "Blood Pressure Change"

2. "Exergame" OR " Exergames" "OR "Exergaming" OR "Exergamings" OR "Active Video Game" OR "Active Video Games" OR "Virtual Reality Exercise" OR "Exercises, Virtual Reality" OR "Exercise, Virtual Reality" OR "Virtual Reality Exercises" OR "Active-Video Gaming" OR "Active Video Gaming" OR "Active-Video Gamings" OR "Gaming, Active-Video" OR "Gamings, Active-Video" OR "interactive video game" OR "interactive video games" OR "Somatosensory Games" OR "Motion-controlled video games" OR "Physical activity video games" OR "Movement-based video games" OR "Fitness video games" OR "active screen time game" OR "Dance Revolution" OR "gamercize power stepper" OR "Wii FitTM" OR "Wii" OR "Nintendo Wii" OR "Kinect" OR "interactive bicycle games" OR "dance videogame" OR "Gesture-based Game" OR "Ring Fit Adventure" OR "Super Mario Party" OR "Fitness Boxing" OR "Just Dance" OR "Augmented Reality Gaming" OR "AR Gaming" OR "AR Games" OR "Motion-Sensing Games" OR "Gesture-Controlled Games" OR "Body-Motion Games" OR "Movement-Tracking Games" OR "Posture-Controlled Games" OR "Sensor-Based Games" OR "Kinect-Based Games" OR "Wii MotionPlus Games" OR "PlayStation Move Games" OR "VR Fitness Games" OR "AR Fitness Games" OR "Interactive Sports Games" OR "Dance-Based Games" OR "Rhythm-Action Games" OR "Exercise Games" OR "Workout Games" OR "Fitness Gaming" OR "Health-Promoting Games" OR "Physical Activity Games" OR "Active Lifestyle Games" OR "Sports Simulation Games" OR "Dance Fitness Games" OR "Cardio Gaming" OR "Strength-Training Games" OR "Balance-Training Games" OR "Rehabilitation Games" OR "Therapeutic Exergames" OR "Xbox Kinect" OR "PlayStation Move" OR "Oculus Fitness Games" OR "HTC Vive Fitness" OR "Zumba Video Game" OR "Wii Sports" OR "Wii Sports Resort" OR "Kinect Sports" OR "Kinect Adventures" OR "Sports Champions" OR "Pokémon Go" OR "Ingress" OR "Eyetoy Games" OR "Nintendo Switch Fitness Games" OR "Fitness Boxing 2" OR "Dance Central" OR "Physical Activity-Enhancing Video Games" OR "Active Gaming Interventions" OR "Video Game-Based Exercise" OR "Digital Health Games" OR "Interactive Screen-Based Activity" OR "Exergaming Systems" OR "Active Video Game Play" OR "Gaming for Fitness" OR "Exercise Videogames" OR "Health-Oriented Gamers" OR "Movement-Required Games" OR "Active Games" OR "Interactive Gaming" OR "Motion Games" OR "Play-and-Move Games" OR "Get-Up-and-Go Games" OR "Active Screen Time" OR "Gaming with Movement" OR "Exercise-Based Video Games" OR "Sports Active Games" OR "Gesture Games" OR "Kinetic Games" OR "AR Gaming" OR "VR Fitness" OR "Motion-Sensing Game"

1 AND 2

**Studies identified from relevant systematic reviews and meta-analysis** (21)

| **Identified from:**  **(Author, year)** | **Study**  **(First author, year)** | **Reference** |
| --- | --- | --- |
| Gao et al, 2015 | AJ Daley, 2009 | Daley, A. J. (2009). Can exergaming contribute to improving physical activity levels and health outcomes in children?. *Pediatrics*, *124*(2), 763-771. |
| Gao et al, 2015 | Penko AL et al, 2010 | Penko, A. L., & Barkley, J. E. (2010). Motivation and physiologic responses of playing a physically interactive video game relative to a sedentary alternative in children. *Annals of Behavioral Medicine*, *39*(2), 162-169. |
| Gao et al, 2015 | Mellecker RR et al, 2008 | Mellecker, R. R., & McManus, A. M. (2008). Energy expenditure and cardiovascular responses to seated and active gaming in children. *Archives of pediatrics & adolescent medicine*, *162*(9), 886-891. |
| Gao et al, 2015 | Perron RM et al, 2012 | Perron, R. M., Graham, C. A., & Hall, E. E. (2012). Comparison of physiological and psychological responses to exergaming and treadmill walking in healthy adults. *GAMES FOR HEALTH: Research, Development, and Clinical Applications*, *1*(6), 411-415. |
| Gao et al, 2015 | Straker LM et al, 2007 | Straker, L., & Abbott, R. (2007). Effect of screen-based media on energy expenditure and heart rate in 9-to 12-year-old children. *Pediatric exercise science*, *19*(4), 459-471. |
| Gao et al, 2015 | Murphy EC-S et al, 2009 | Murphy, E. C., Carson, L., Neal, W., Baylis, C., Donley, D., & Yeater, R. (2009). Effects of an exercise intervention using Dance Dance Revolution on endothelial function and other risk factors in overweight children. *International Journal of Pediatric Obesity*, *4*(4), 205-214. |
| Gao et al, 2015 | Gao Z et al, 2014 | Gao, Z., & Xiang, P. (2014). Effects of exergaming based exercise on urban children’s physical activity participation and body composition. *Journal of Physical Activity and Health*, *11*(5), 992-998. |
| Gao et al, 2015 | Azevedo LB et al, 2014 | Azevedo, L. B., Burges Watson, D., Haighton, C., & Adams, J. (2014). The effect of dance mat exergaming systems on physical activity and health–related outcomes in secondary schools: results from a natural experiment. *BMC public health*, *14*, 1-13. |
| Lourenço et al, 2022 | Brito-Gomes et al, 2018 | Brito-Gomes, J. L. D., Perrier-Melo, R. J., Brito, A. D. F., & Costa, M. D. C. (2018). Active videogames promotes cardiovascular benefits in young adults? Randomized controlled trial. *Revista Brasileira de Ciências do Esporte*, *40*(1), 62-69. |
| Lourenço et al, 2022 | Warburton, D. E et al, 2007 | Warburton, D. E., Bredin, S. S., Horita, L. T., Zbogar, D., Scott, J. M., Esch, B. T., & Rhodes, R. E. (2007). The health benefits of interactive video game exercise. *Applied Physiology, Nutrition, and Metabolism*, *32*(4), 655-663. |
| Lourenço et al, 2022 | Moholdt, T et al, 2017 | Moholdt, T., Weie, S., Chorianopoulos, K., Wang, A. I., & Hagen, K. (2017). Exergaming can be an innovative way of enjoyable high-intensity interval training. *BMJ open sport & exercise medicine*, *3*(1). |
| Moller et al, 2023 | Best J. R, 2013 | Best J. R. (2013). Exergaming in Youth: Effects on Physical and Cognitive Health. *Zeitschrift fur Psychologie*, *221*(2), 72–78. https://doi.org/10.1027/2151-2604/a000137 |
| Moller et al, 2023 | Gao, Z et al, 2016 | Gao, Z., Lee, J. E., Pope, Z., & Zhang, D. (2016). Effect of Active Videogames on Underserved Children's Classroom Behaviors, Effort, and Fitness. *Games for health journal*, *5*(5), 318–324. https://doi.org/10.1089/g4h.2016.0049 |
| Moller et al, 2023 | Bosch, P. R et al, 2012 | Bosch, P. R., Poloni, J., Thornton, A., & Lynskey, J. V. (2012). The heart rate response to nintendo wii boxing in young adults. *Cardiopulmonary physical therapy journal*, *23*(2), 13–29. |
| Moller et al, 2023 | Bock, B. C et al, 2019 | Bock, B. C., Dunsiger, S. I., Ciccolo, J. T., Serber, E. R., Wu, W. C., Tilkemeier, P., Walaska, K. A., & Marcus, B. H. (2019). Exercise Videogames, Physical Activity, and Health: Wii Heart Fitness: A Randomized Clinical Trial. *American journal of preventive medicine*, *56*(4), 501–511. https://doi.org/10.1016/j.amepre.2018.11.026 |
| Moller et al, 2023 | Duncan, M. J et al, 2011 | Duncan, M. J., Birch, S., Woodfield, L., & Hankey, J. (2011). Physical activity levels during a 6-week, school-based, active videogaming intervention using the gamercize power stepper in British children. *Medicina Sportiva*, *15*(2). |
| Moller et al, 2023 | Fu, Y et al, 2018 | Fu, Y., & Burns, R. D. (2018). Effect of an Active Video Gaming Classroom Curriculum on Health-Related Fitness, School Day Step Counts, and Motivation in Sixth Graders. *Journal of physical activity & health*, *15*(9), 644–650. https://doi.org/10.1123/jpah.2017-0481 |
| Moller et al, 2023 | Gao, Z et al, 2019 | Gao, Z., Lee, J. E., Zeng, N., Pope, Z. C., Zhang, Y., & Li, X. (2019). Home-Based Exergaming on Preschoolers' Energy Expenditure, Cardiovascular Fitness, Body Mass Index and Cognitive Flexibility: A Randomized Controlled Trial. *Journal of clinical medicine*, *8*(10), 1745. https://doi.org/10.3390/jcm8101745 |
| Moller et al, 2023 | Howie, E. K et al, 2016 | Howie, E. K., Campbell, A. C., & Straker, L. M. (2016). An active video game intervention does not improve physical activity and sedentary time of children at-risk for developmental coordination disorder: a crossover randomized trial. *Child: care, health and development*, *42*(2), 253–260. https://doi.org/10.1111/cch.12305 |
| Moller et al, 2023 | Maddison, R. et al, 2011 | Maddison, R., Foley, L., Ni Mhurchu, C., Jiang, Y., Jull, A., Prapavessis, H., Hohepa, M., & Rodgers, A. (2011). Effects of active video games on body composition: a randomized controlled trial. *The American journal of clinical nutrition*, *94*(1), 156–163. https://doi.org/10.3945/ajcn.110.009142 |
| Moller et al, 2023 | Maloney, A. E et al, 2012 | Maloney, A. E., Threlkeld, K. A., & Cook, W. L. (2012). Comparative Effectiveness of a 12-Week Physical Activity Intervention for Overweight and Obese Youth: Exergaming with "Dance Dance Revolution". *Games for health journal*, *1*(2), 96–103. https://doi.org/10.1089/g4h.2011.0009 |
