# Supplementary material for: Role of Active Video Games in Blood Pressure Management Among Children and Young Adults: Systematic Review and Meta-Analysis
Source: J Med Internet Res. 2025 Aug 19;27:e75000. doi: 10.2196/75000 (PMC12381676; doi:10.2196/75000)
Supplement: Multimedia Appendix 2 [file jmir-v27-e75000-s002.docx]

Appendix S2. Cochrane Risk of Bias tool 2.0 for the included controlled trials.


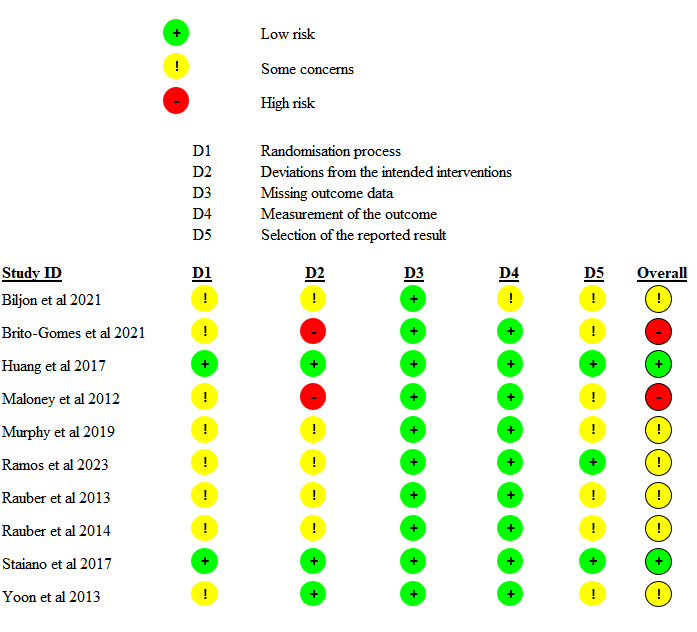

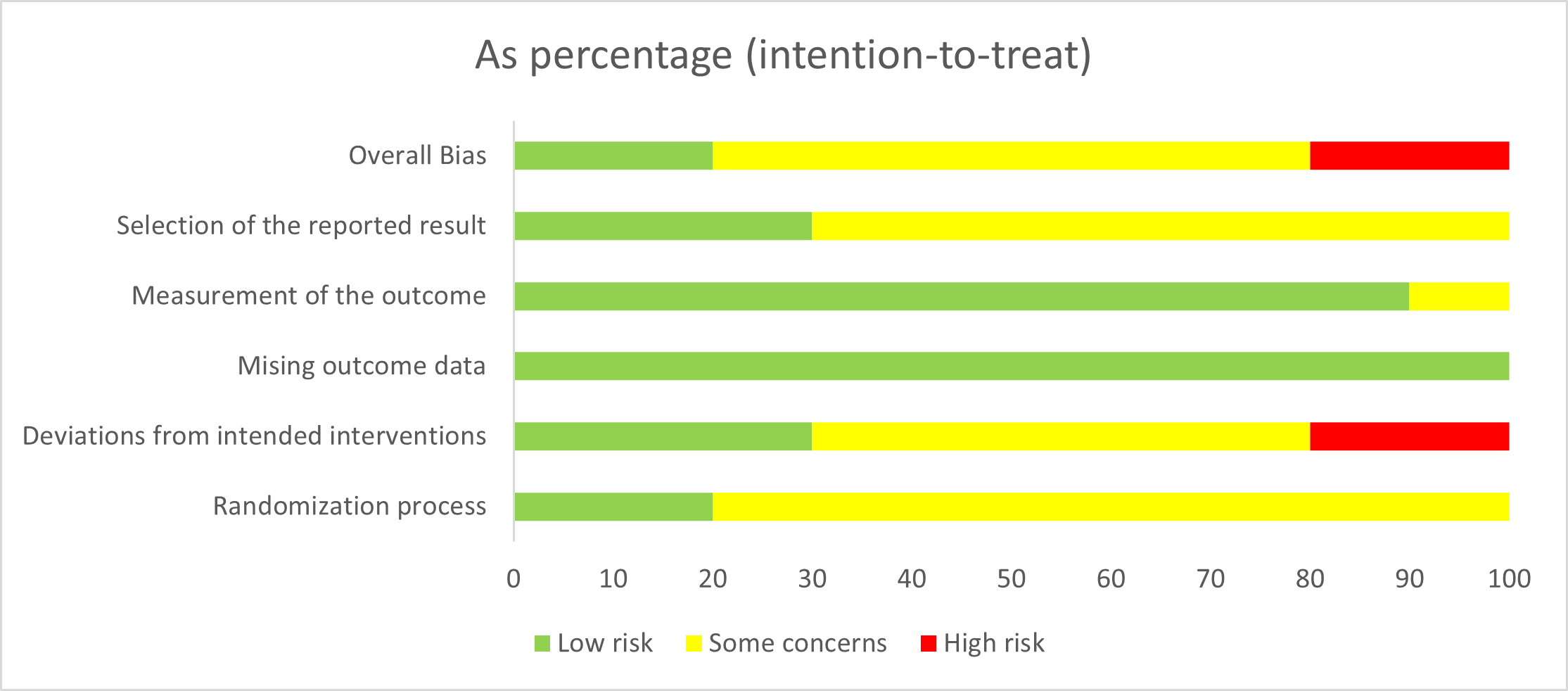


Appendix S3. The Methodological Items for Non-Randomized Studies (MINORS) tools for the included pre-post trials.

| Study | Item number | | | | | | | | Score |
| --- | --- | --- | --- | --- | --- | --- | --- | --- | --- |
|  | 1 | 2 | 3 | 4 | 5 | 6 | 7 | 8 |  |
| Barbosa et al (2021) | 2 | 2 | 1 | 1 | 0 | 1 | 0 | 0 | 7/16 |
| Bethea et al (2012) | 2 | 2 | 1 | 1 | 0 | 2 | 0 | 0 | 8/16 |
| Carrasco et al (2013) | 2 | 1 | 1 | 1 | 0 | 1 | 2 | 0 | 8/16 |
| Brito-Gomes et al (a) (Feb, 2019) | 2 | 2 | 1 | 1 | 0 | 0 | 2 | 2 | 10/16 |
| Brito-Gomes et al (b) (Feb, 2019) | 2 | 2 | 1 | 1 | 0 | 1 | 2 | 2 | 11/16 |
| Brito-Gomes et al (July, 2019) | 2 | 2 | 1 | 1 | 0 | 0 | 2 | 0 | 8/16 |
| Roopchand-Martin et al (2015) | 2 | 2 | 1 | 1 | 0 | 1 | 0 | 0 | 7/16 |

Item 1, A clearly stated aim

Item 2, Inclusion of consecutive patients

Item 3, Prospective collection of data

Item 4, Endpoints appropriate to the aim of the study

Item 5, Unbiased assessment of the study endpoint

Item 6, Follow-up period appropriate to the aim of the study

Item 7, Loss to follow up less than 5%

Item 8, Prospective calculation of the study size

*0: Not reported, 1: Reported but inadequate, 2: Reported and adequate
