# Supplementary material for: Role of Active Video Games in Blood Pressure Management Among Children and Young Adults: Systematic Review and Meta-Analysis
Source: J Med Internet Res. 2025 Aug 19;27:e75000. doi: 10.2196/75000 (PMC12381676; doi:10.2196/75000)
Supplement: Multimedia Appendix 3 [file jmir-v27-e75000-s003.docx]

**Appendix S4.** Grading of Recommendations Assessment, Development and Evaluation

GRADE initially assumes the evidence quality to be high, which is then evaluated for potential downgrading by researchers based on five domains: study limitations, inconsistency of results, indirectness of evidence, imprecision, and publication bias. **Study limitation:** Downgraded if there was existence of risk of bias that could affect study results; **Inconsistency of results:** Downgraded if substantial heterogeneity that could not be explained (e.g., by sensitivity analysis, subgroup analysis or meta-regression, etc.) could affect result interpretation; **Indirectness of evidence:** Downgraded if differences in population, interventions, outcome measures or comparisons existed that could limit the generalized ability of results to our target population and/or outcomes. **Imprecision:** Downgraded if studies had small sample size and were presented with a wide confidence interval around the effect estimate, signifying imprecision of results; **Publication bias:** Downgraded if publication bias was found as revealed through Egger’s tests (*P* -value < 0.05) and visual examination using funnel plots symmetry.

In this study, the downgrading criteria for GRADE are set as follows:

Study limitation: More than 50% of the studies were at high risk in RoB assessment or MINORS scores of more than half of the studies were less than 8.

Inconsistency of results: There is heterogeneity (I² > 50% or *P* for Q - statistic < 0.05).

Indirectness of evidence: If there are differences in the population, intervention measures, outcome measurements, or comparisons, which may limit the generalizability of the results to the target population and/or outcomes, the evidence is downgraded.

Imprecision: The number of participants is less than 500, the confidence interval width exceeded 0.5, or the sensitivity analysis failed.

Publication bias: The p - value of the Egger’s test < 0.05 and the results change significantly after applying trim-and-fill method.

Certainty of evidence are then classified into high, moderate, low or very low, depending on whether there is a need of downgrading the certainty. Implication of evidence certainty grades on further research are as follows:

⊕⊕⊕⊕ High: Further research is unlikely to change the confidence in the estimate of effect.

⊕⊕⊕⊖ Moderate: Further research is likely to have an important impact on the confidence in the estimate of effect and may change the estimate.

⊕⊕⊖⊖ Low: Further research is very likely to have an important impact on the confidence in the effect estimate and is likely to change the estimate.

⊕⊖⊖⊖ Very low: Any estimate of effect is very uncertain.

|  |  | Study Limitation | Inconsistency of Results | Indirectness of Evidence | Imprecision | Publication Bias | Overall |
| --- | --- | --- | --- | --- | --- | --- | --- |
| Participants aged 6 to 25 | SBP | No | Yes | No | No | Yes | Low |
|  | DBP | No | No | No | Yes | No | Moderate |
|  |  |  |  |  |  |  |  |
| Children under 18 | SBP | No | Yes | No | Yes | No | Low |
|  | DBP | No | No | No | Yes | No | Moderate |
