# Supplementary material for: Role of Active Video Games in Blood Pressure Management Among Children and Young Adults: Systematic Review and Meta-Analysis
Source: J Med Internet Res. 2025 Aug 19;27:e75000. doi: 10.2196/75000 (PMC12381676; doi:10.2196/75000)

**Figure S1.** The effect of AVGs on SBP from controlled trials.


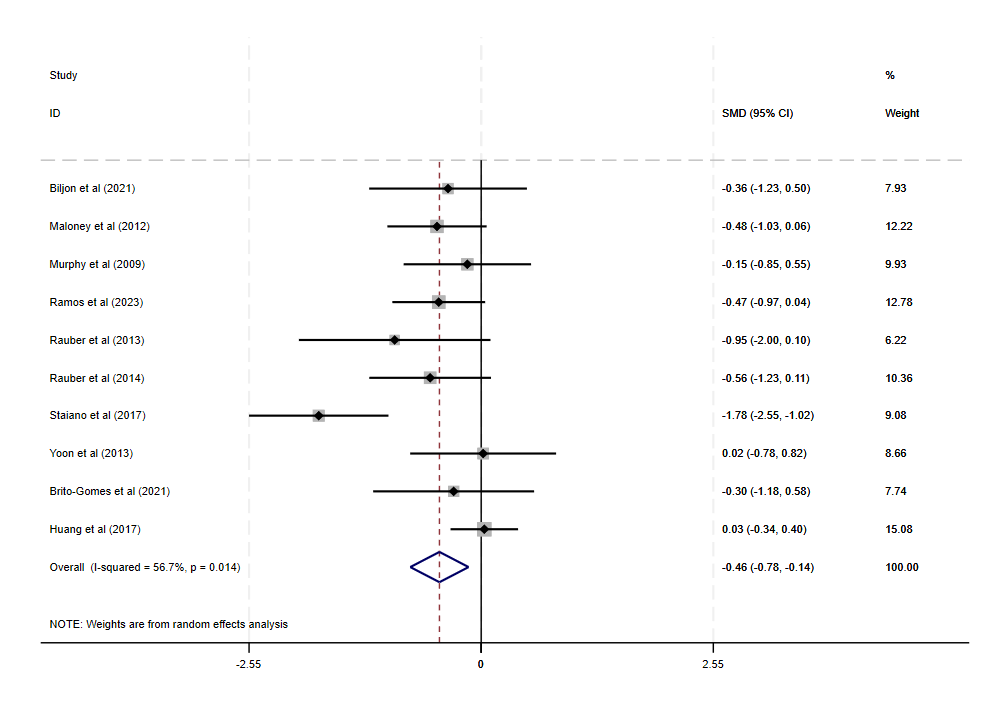


**Figure S2.** The effect of AVGs on DBP from controlled trials.


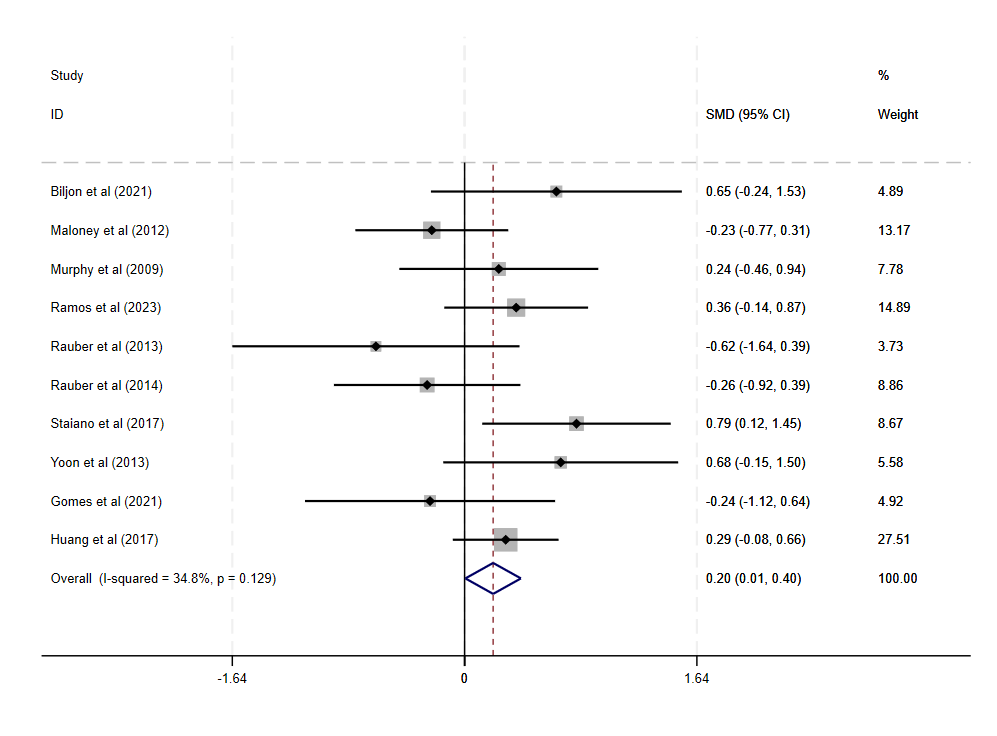


**Figure S3.** Effect of AVGs on SBP in trials with blood pressure measurements taken in laboratory settings.


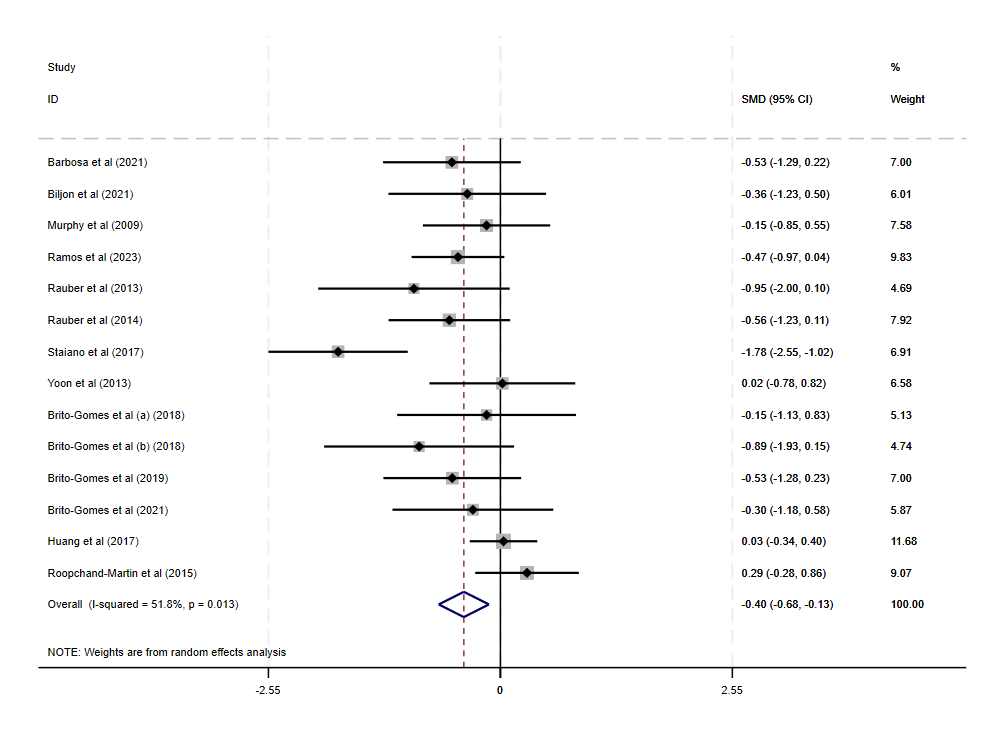


**Figure S4.** Effect of AVGs on DBP in trials with blood pressure measurements taken in laboratory settings


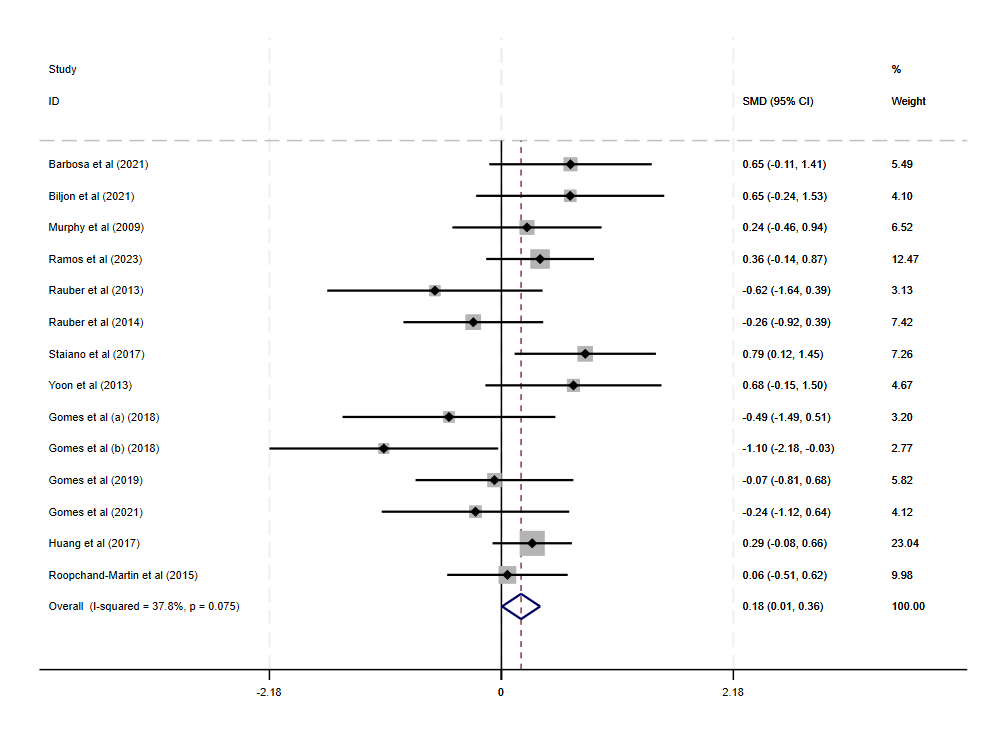

Supplement: Multimedia Appendix 4 [file jmir-v27-e75000-s004.docx]
