# Supplementary material for: Role of Active Video Games in Blood Pressure Management Among Children and Young Adults: Systematic Review and Meta-Analysis
Source: J Med Internet Res. 2025 Aug 19;27:e75000. doi: 10.2196/75000 (PMC12381676; doi:10.2196/75000)
Supplement: Multimedia Appendix 5 [file jmir-v27-e75000-s005.docx]

Appendix S5. The results of funnel plots

| 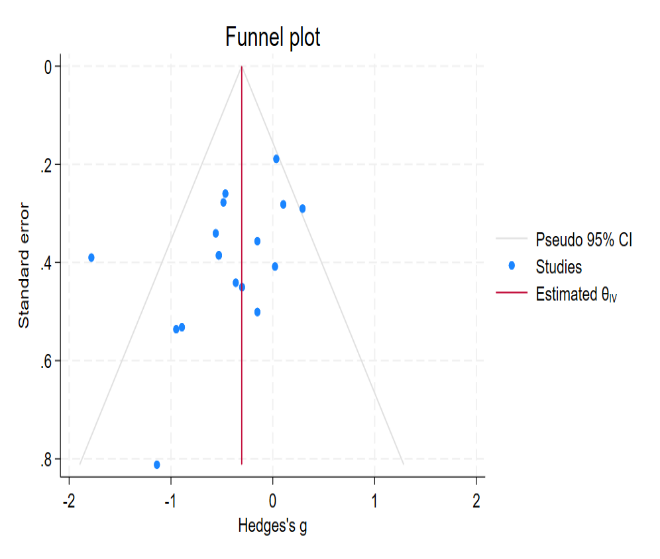 | 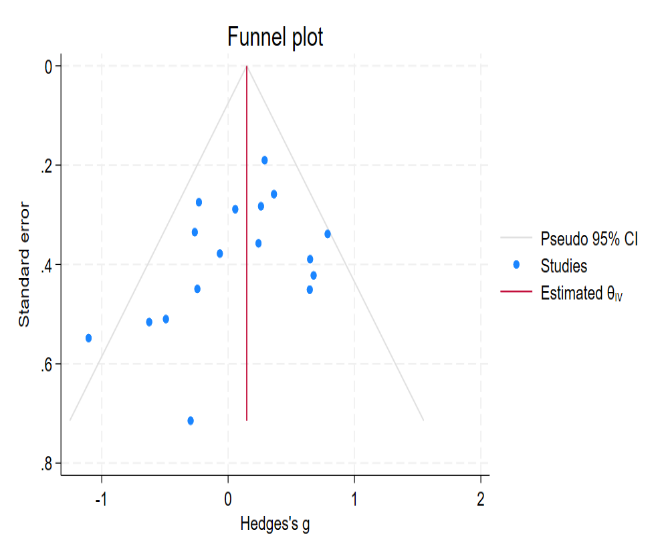 |
| --- | --- |
| SBP in young adults (18 – 25 years) | DBP in young adults (18 – 25 years) |
| 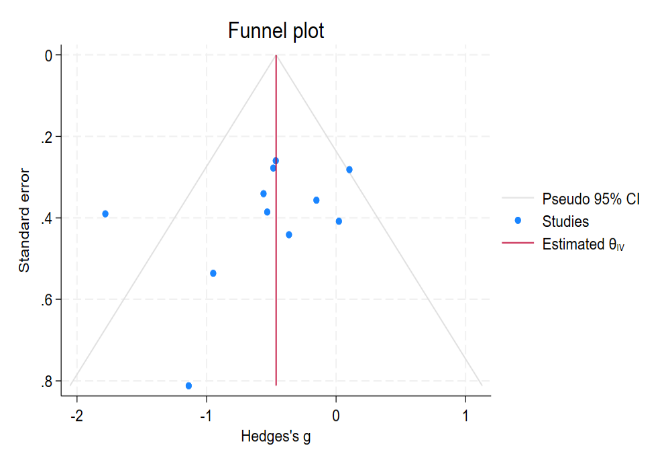 | 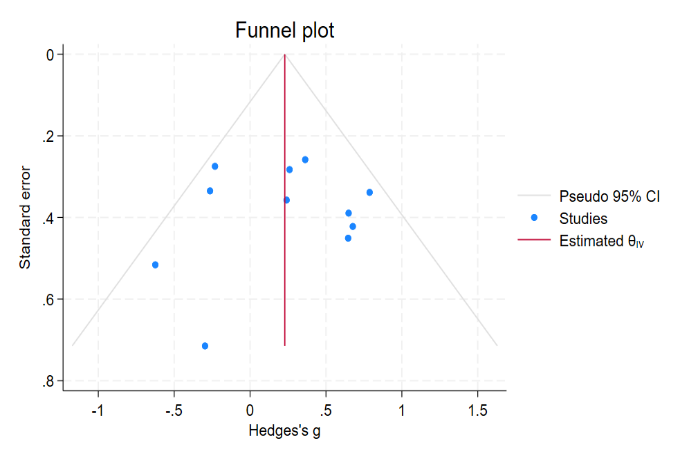 |
| SBP in children (<18 years) | DBP in children (<18 years) |
