# Supplementary figures and images for: Role of Active Video Games in Blood Pressure Management Among Children and Young Adults: Systematic Review and Meta-Analysis
Source: J Med Internet Res. 2025 Aug 19;27:e75000. doi: 10.2196/75000 (PMC12381676; doi:10.2196/75000)

**Appendix S6.** The results of trim-and-fill method

| 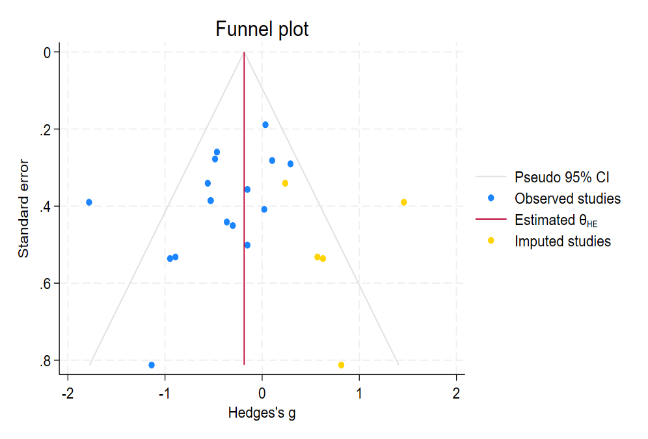 | 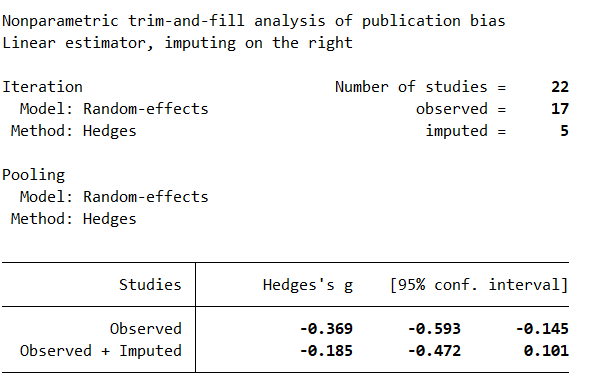 |
| --- | --- |
| Funnel plot | Effect size |

Supplement: Multimedia Appendix 6 [file jmir-v27-e75000-s006.docx]
